# Lymphatic Involvement in the Disappearance of Steroidogenic Cells from the Corpus Luteum during Luteolysis

**DOI:** 10.1371/journal.pone.0088953

**Published:** 2014-02-20

**Authors:** Hironori Abe, Mohamad Omar Al-zi’abi, Fumio Sekizawa, Tomas J. Acosta, Dariusz J. Skarzynski, Kiyoshi Okuda

**Affiliations:** 1 Laboratory of Reproductive Physiology, Graduate School of Environmental and Life Science, Okayama University, Okayama, Japan; 2 Sekizawa Animal Clinic, Tochigi, Japan; 3 Department of Reproductive Immunology and Pathology, Institute of Animal Reproduction and Food Research, Polish Academy of Sciences, Olsztyn, Poland; Imperial College London, United Kingdom

## Abstract

In mammals, the corpus luteum (CL) is an essential endocrine gland for the establishment and maintenance of pregnancy. If pregnancy is not established, the CL regresses and disappears rapidly from the ovary. A possible explanation for the rapid disappearance of the CL is that luteal cells are transported from the ovary via lymphatic vessels. Here, we report the presence of cells positive for 3β-hydroxysteroid dehydrogenase (3β-HSD), an enzyme involved in progesterone synthesis, in the lumen of lymphatic vessels at the regressing luteal stage and in the lymphatic fluid collected from the ovarian pedicle ipsilateral to the regressing CL. The 3β-HSD positive cells were alive and contained lipid droplets. The 3β-HSD positive cells in the lymphatic fluid were most abundant at days 22–24 after ovulation. These findings show that live steroidogenic cells are in the lymphatic vessels drained from the CL. The outflow of steroidogenic cells starts at the regressing luteal stage and continues after next ovulation. The overall findings suggest that the complete disappearance of the CL during luteolysis is involved in the outflow of luteal cells from the CL via ovarian lymphatic vessels.

## Introduction

In mammals, the corpus luteum (CL) formed after ovulation is an essential endocrine organ for the establishment and maintenance of pregnancy [1]. If pregnancy is not established, the CL regresses rapidly. The regression of the CL is essential to reset the ovarian cycle, so that mammals obtain another chance to become pregnant.

CL regression (luteolysis) consists of two phases, functional luteolysis and structural luteolysis [2]. Functional luteolysis is defined as declining in progesterone (P4) production. Structural luteolysis is characterized by a decrease in the volume of the CL due to loss of luteal cells. In cows, structural luteolysis is induced by uterine prostaglandin F2α (PGF2α), it takes 4–5 days to reduce the size of the CL [3]. This disappearance of CL tissue during structural luteolysis is generally explained by apoptosis of luteal cells and phagocytosis by intraluteal macrophages [2], [4], [5]. In agreement with this concept, the number of macrophages increases at the regressing luteal stage (Penny *et al.*, 1999). On the other hand, the volume of the CL decreases to less than half of its original size in 24 h after administration of PGF2α on Day 10 post ovulation [6]. However, the number of macrophages observed within the regressing CL during spontaneous and PGF2α-induced luteolysis is almost same [7]. Therefore, the mechanisms involved in the rapid disappearance of CL from the ovary have been a mystery for reproductive scientists.

The lymphatic vascular system, which is considered the body’s second circulation system, is essential for transporting interstitial fluid, macromolecules (proteins, lipids) and cells [8]. The ovary has a rich network of lymphatic vessels. The lymphatic system has been suggested to be associated with folliculogenesis [9]. The CLs of primates and cows have lymphatic vessels [10], [11]. However, the function of the lymphatic vasculature in the CL is unclear.

In most human cancers, the lymphatic vasculature serves as the primary route for the metastatic spread of tumor cells to regional lymphatic nodes [8]. In a preliminary experiment, we found large lymphatic vessels near degenerated arterioles in the regressing CL. Therefore, we hypothesized that during structural luteolysis of bovine CL, a large number of luteal cells flow out of the CL through the lymphatic vessels, similar to the flow of cancer cells in lymphatic vessels. The purpose of the present study was to test the above hypothesis. If confirmed, it could provide a novel explanation for the rapid decrease in size of the CL during structural lutolysis.

## Materials and Methods

### Ethics Statement

In this study, we did not perform any animal experiments. The ovaries were collected from non-pregnant and pregnant (days 120–180 of pregnancy) Holstein cows at local abattoir (Tsuyama Meat Center) in accordance with protocols approved by local institutional animal care. The gestational ages were determined from fetal crown-rump length and classified as days 60–90 (6–20 cm), days 120–180 (25–45 cm) and days 210–270 (50–80 cm) [12]. All the samples and data analyzed in the present study were obtained with the permission of the above center.

### Collection of Ovary with CL and Lymphatic Fluid

**Figure 1:**
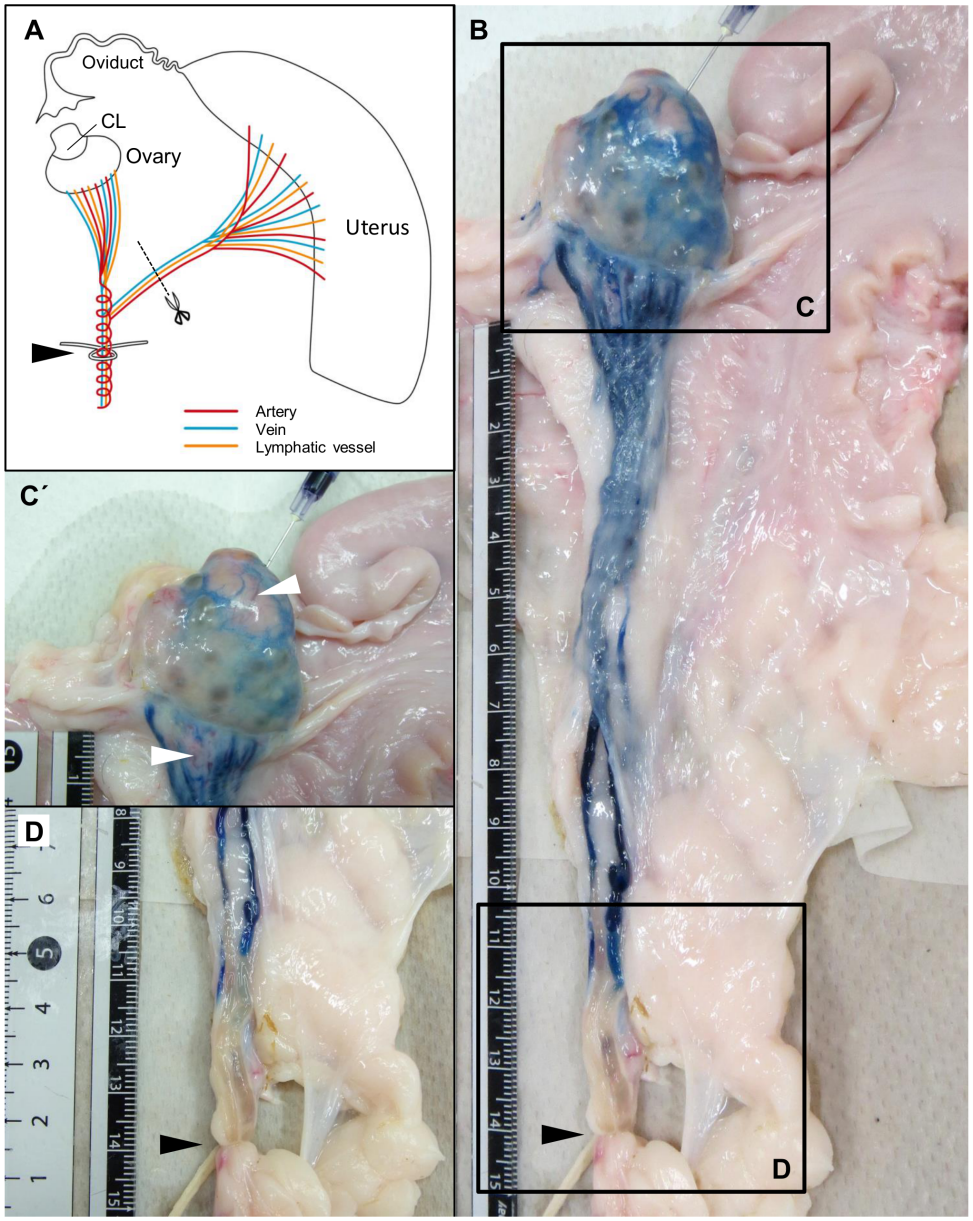
Anatomy of the ovarian lymphatic vessels. A: The broad ligament has at least two lymphatic routes, one from the ovary and the other from the uterus. To collect lymphatic fluids, the lymphatic vessels were ligated at 15(black arrowhead). To avoid mixing ovarian and uterine lymphatic fluids, the lymphatic vessel draining the uterus was cut. B: To confirm that the collected lymphatic fluid was drained from the CL, 2 ml of 0.1% Evans Blue (EB) solution was injected into the CL (C). C: EB injected into the CL drained via lymphatic vessels (D). C: EB was not observed in blood vessels (white arrowheads). D: The lymphatic fluid pooled in the lymphatic vessels was aspirated using a needle and syringe. Black arrowhead shows the ligation point.

The bovine CL and lymphatic fluid drained from the ovary were obtained from a local abattoir in accordance with protocols approved by the local institutional animal care and use committee. In cow, the ovulation occurs randomly in each ovary. Therefore, it is possible that the fresh and regressing CL are in a ovary, or also in each ovary. To identify the source of the cells in the lymphatic fluid, the lymphatic fluid was collected drained from a ovary with only a single CL. The CLs and lymphatic fluid samples were obtained within 30 min after exsanguination and were transported to the laboratory within 1–1.5 h on ice. In cows, ovulation generally occurs every 21 days. Luteal stages were classified as being early (Days 2–3 after ovulation), developing (Days 5–7), mid (Days 8–12), late (Days 15–17), regressing (Days 19–21) [13], [14]. The Days of previous CL were calculated based on the luteal stages of active CL on the contralateral ovary. For collecting the lymphatic fluid, the lymphatic vessels were, immediately after exsanguination ligated at 15 cm from ovarian hilus to allow accumulation of lymphatic fluid (Fig. 1B). Uterine blood and lymphatic vessels were cut immediately after ligation. Then, lymphatic vessels were dissected from connective tissue and lymphatic fluid accumulated in vessels was collected using a needle and syringe. To confirm that lymphatic vessels were drained from the CL, 2 ml of 0.1% Evans Blue staining solution was injected directly into the CL (Fig. 1).

### Immunohistochemistry

CLs were fixed with 4% (v/v) paraformaldehyde (PFA) in PBS. 4 µm sections were mounted on glass slides pre-coated with silane (S3003; Dako, Glostrup, Denmark), deparaffined and rehydrated. Antigen retrieval was achieved by Tris-EDTA buffer pH 9.0 using microwave for 15 min at 600 W. Sections were immersed in methanol with 3% (v/v) H2O2 for 30 min and incubated with 10% (v/v) normal horse serum (MP-7500; Vector Laboratories Inc, Burlingame, CA, USA) for blocking. Then, sections were incubated with LYVE-1 antibody (ab33682; abcam, Cambridge, UK) as a specific marker of lymphatic endothelial cells [10], [15], [16] diluted at 1∶4000 with PBS for 1 h at room temperature (RT). Negative control sections were incubated with normal rabbit serum diluted by PBS. Subsequently, sections were incubated with ImmPRESS UNIVERSAL reagent, anti-mouse/rabbit Ig (MP-7500; Vector Laboratories Inc, Burlingame, CA, USA) for 30 min according to the manufacturer’s instructions. The staining was visualized with 0.05% (w/v) 3,3′-diaminobenzidine (343-00901; Dojindo, Kumamoto, Japan) containing 0.01% (v/v) H2O2 and then counter-stained with hematoxylin. Bright field images were captured using FSX100 (Olympus, Tokyo, Japan) and merged using cellSens (Olympus, Tokyo, Japan).

### Double Fluorescent Immunohistochemistry

4 µm sections were mounted on glass slides pre-coated with silane, deparaffined and rehydrated. Antigen retrieval was achieved by Tris-EDTA buffer pH 9 using microwave for 15 min at 600 W then sections were incubated with 10% (v/v) normal horse serum for blocking. Sections were incubated with LYVE-1 antibody diluted at 1∶2000 with PBS and 3β-HSD antibody (ab75710; Abcam, Cambridge, UK) as a steroidogenic cell marker diluted 1∶1000 with PBS for 1 h at RT. Negative control sections were incubated with normal rabbit serum and normal mouse serum diluted by PBS. Subsequently, the sections were incubated with Alexa Fluor 488 goat anti-rabbit IgG antibody (A-11008; Life Technologies, Carlsbad, CA, USA) and Alexa Fluor 594 goat anti-mouse IgG antibody (A-11005 Life Technologies, Carlsbad, CA, USA) for 1 h at RT. Nuclei were visualized using ProLong Gold including DAPI (P36935; Life Technologies, Carlsbad, CA, USA). Fluorescent images were captured using FSX100 and merged using cellSens (Olympus, Tokyo, Japan).

### Smear of Lymphatic Fluid and Staining

To determine the viability of cells found in the lymphatic fluid, trypan blue solution was diluted in lymphatic fluid to 0.12% finally. Immediately, the cells in the lymphatic fluid were observed using light field microscope. The smears of the lymphatic fluid for each staining were performed according to the general biopsy manual. The lymphatic fluids smeared on glass slides pre-coated with silane were dried immediately and rehydrated with PBS. Subsequently the smears were dipped in 4% (v/v) PFA in PBS for 10 min at RT.

For fluorescent staining, the smears were incubated in 0.1% triton X-100 solution for 5 min. To inhibit non-specific staining, the smears were incubated with 10% (v/v) normal horse serum for 30 min. To demonstrate the presence of luteal cells in lymphatic fluid, the smear was incubated with 3β-HSD antibody diluted at 1∶500 with PBS for 30 min at RT. Then the smear was incubated with Alexa Fluor 488 Goat anti-mouse IgG (A-11001; Life Technologies, Carlsbad, CA, USA) diluted at 1∶1000 with PBS for 30 min at RT. The smear was incubated with Bodipy 493/503 (D-3922; Life Technologies, Carlsbad, CA, USA) used for lipid droplets-staining [17] diluted at 1∶200 with PBS for 20 min at RT. Nuclei of all cells in smear were visualized using ProLong Gold including DAPI (P36935; Life Technologies, Carlsbad, CA, USA)then examined using FSX100. In this staining, bovine uterine stromal cultured cells were used as negative control.

### Quantification of Luteal Cells in Lymphatic Fluid

To calculate the ratio of luteal cells to the whole cells in lymphatic fluid smear, 3 fields per a smear were selected randomly. The cells positively immunostained with 3β-HSD antibody and DAPI nuclear staining coinciding were counted by three independent observers. The percentage of the luteal cells in lymphatic fluid was calculated as X/Y×100, where X was the total number of immunostaining positive cells and Y was the total number of DAPI positive cells in the fields selected.

### Statistical Analysis

The statistical significance of differences in percentage of luteal cells in lymphatic fluids were assessed by analysis of variance (ANOVA) followed by a Fisher protected least significant difference procedure (PLSD) as a multiple comparison test and Bonferroni correction.

## Results

### Localization of Lymphatic Vessels and Steroidogenic Cells

**Figure 2:**
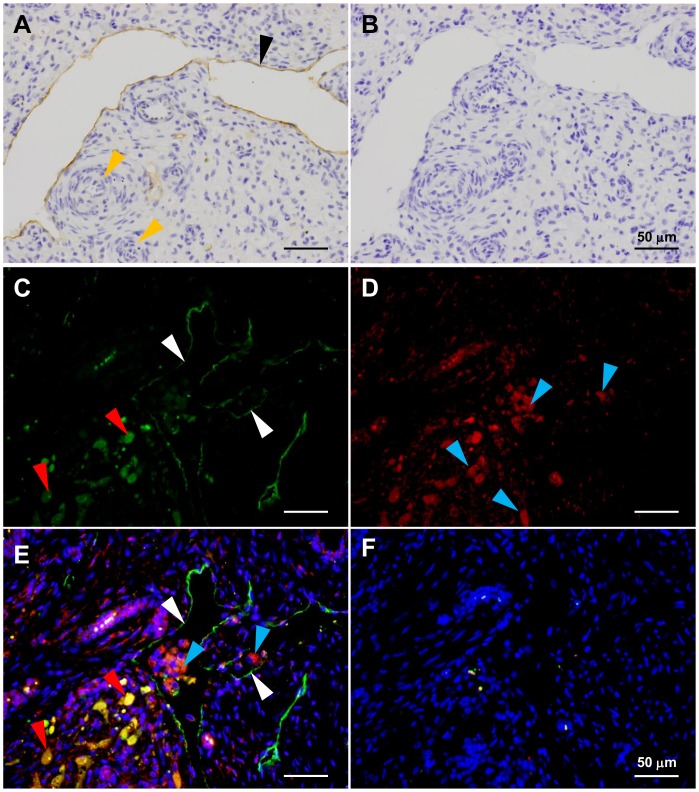
Immunohistochemical staining of the regressing bovine CL tissue. A: Large lymphatic vessels (black arrowheads) were observed around arterioles (yellow arrowheads). B: Negative control. C: Lymphatic vessels were visualized by LYVE-1 antibody and goat anti rabbit IgG antibody labeled with Alexa 488 (white arrowheads). D: Steroidogenic cells were detected by 3β-HSD antibody and goat anti mouse IgG antibody labeled with Alexa 594 (blue arrowheads). E: Many 3β-HSD positive cell were observed in the lumen of LYVE-1 positive vessel (merge C and D). LYVE-1 antibody positively reacted with 3β-HSD positive cells (red arrowheads). F: Negative control. Nuclei were stained using DAPI. All scale bars, 50 µm.

In the regressing CL, large lymphatic vessels were detected near arterioles by staining with LYVE-1 antibody (Fig. 2A). Many 3β-HSD positive cells were found in the lumen of lymphatic vessels (Fig. 2E). LYVE-1 antibody reacted with 3β-HSD positive cells (Figs. 2C, D and E).

### Characterization of Cells in the Lymphatic Fluid

**Figure 3:**
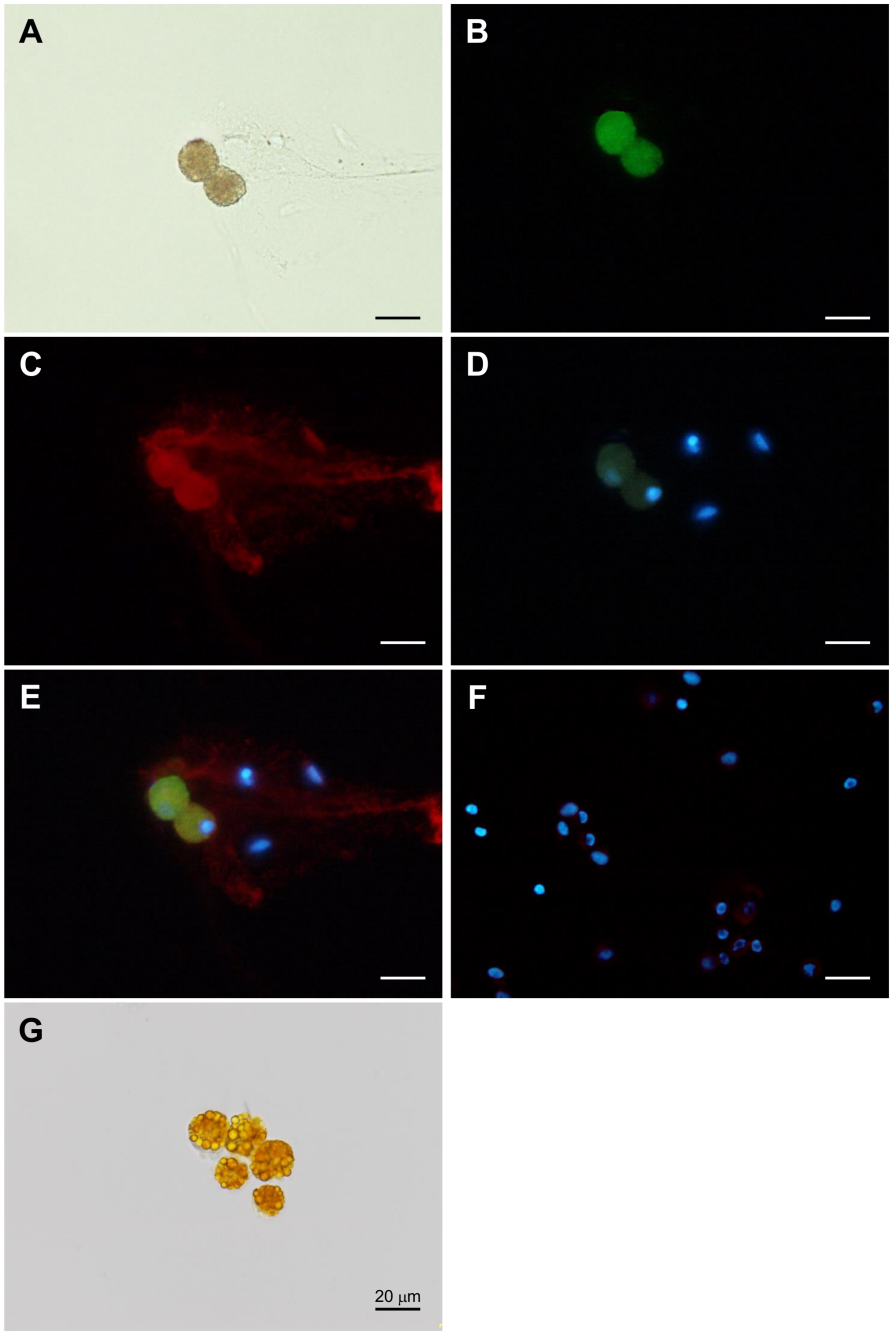
Staining of lymphatic fluid smear. A: Bright field image of the cells in lymphatic fluid. B: Lipid droplets in the cells were stained by Bodipy 493/503. C: The same cells show a positive signal for 3β-HSD. D: The nuclei in the cells were visualized using DAPI. E: Merge image of B, C and D. The nucleus was located by marginal cell membrane (white arrowhead). F: Cultured bovine endometrial stromal cells were used as negative control. G: Many lipid droplets were observed as granules in cytoplasm (black arrowhead). Most of the cells in the lymphatic fluids were not stained by trypan blue solution. All scale bars, 20 µm.

The volumes of lymphatic fluid collected from lymphatic vessels ranged from 10 to 200 µl. Most lymphatic fluid was accumulated within 15 min after ligation. The color of the fluid was variously as clear and yellow. Many yellow cells were found in the lymphatic fluid (Fig. 3A). Their sizes of those cells were about 20 µm and granules were observed in the cytoplasm (Fig. 3G). The yellow cells positively reacted with Bodipy (493/503) and 3β-HSD antibody (Figs. 3B and C). The nucleus of yellow cells was observed at marginal cell membrane (Fig. 3E). Trypan blue did not stain most of the yellow cells (Figs. 3G and H), indicating that the cells were alive.

### Changes in Ratio of 3β-HSD Positive Cells in Lymphatic Fluid

**Figure 4:**
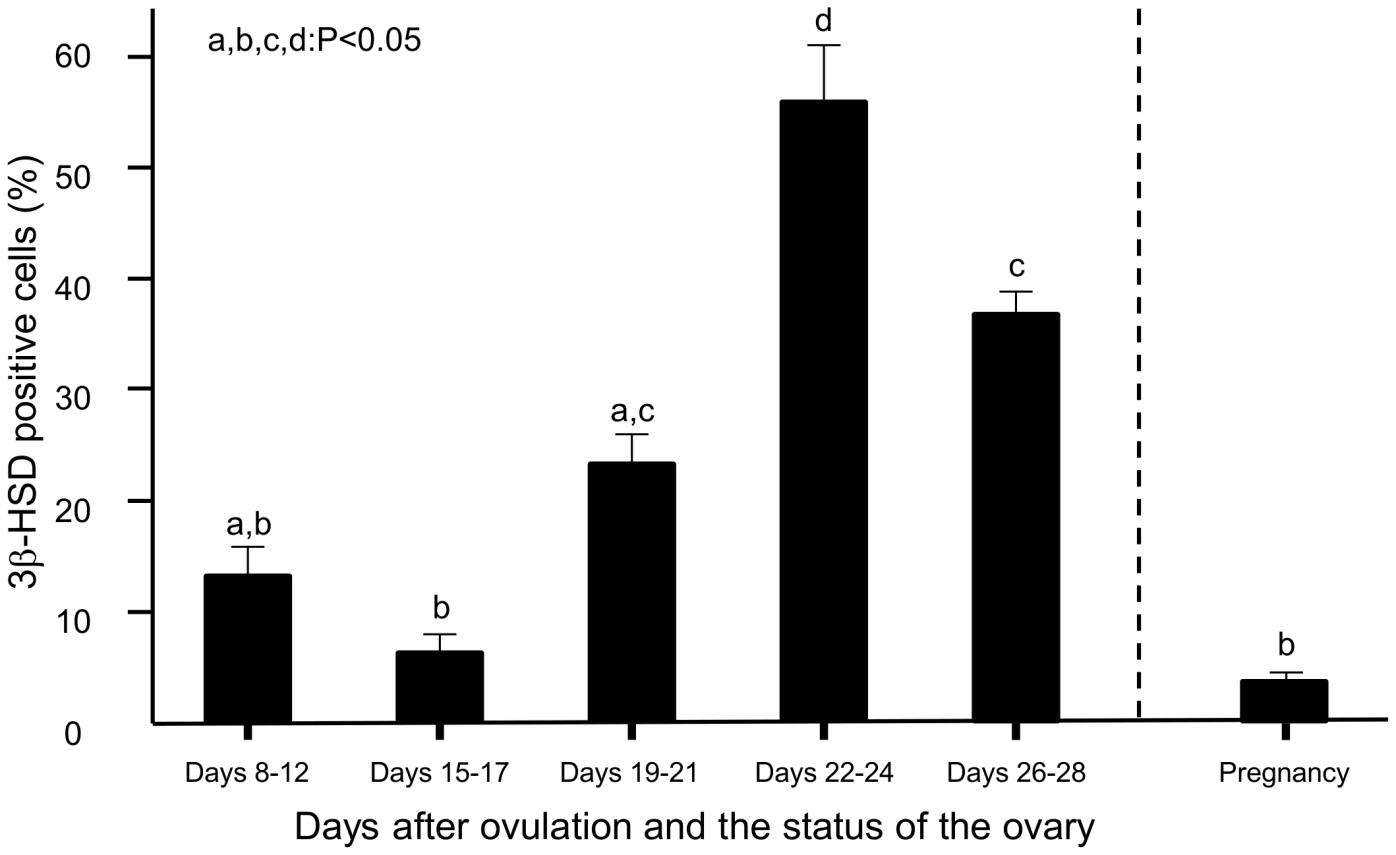
3β-HSD positive cells in the lymphatic fluid during the estrous cycles and pregnancy. Different superscript letters indicate significant difference (P<0.05) compared with the other luteal stages as assessed by ANOVA followed by a Fisher’s protected least significant difference procedure (PLSD) as a multiple comparison test (n = 3/luteal stage). The percentage of 3β-HSD positive cells in the lymphatic fluid significantly increased at days 22–24 after ovulation.

The percentage of 3β-HSD positive cells to whole cells present in lymphatic fluid was significantly (P<0.05) higher at days 22–24 than that at the other luteal stages including mid-pregnancy (Fig. 4). Furthermore, at days 26–28 after ovulation, the ratio of 3β-HSD positive cells was significantly (P<0.05) higher than that at days 8–12 and 15–17 after ovulation (Fig. 4).

## Discussion

The main function of the lymphatic vasculature is to transport fluid, macromolecules and cells including monocytes and lymphocytes from the tissues to the blood circulation [8]. In most cancers, the lymphatic vessels serve as the primary route for the metastatic spread of tumor cells [8]. Thus, lymphatic vessels in the CL may be a route for transporting luteal cells to outside of the CL. In the present study, we used a lymphatic endothelial hyaluronan receptor (LYVE-1) antibody and 3β-HSD antibody, a specific steroidogenic cell marker. Double-fluorescent immunohistochemistry revealed cells that react with 3β-HSD antibody in the lumen of lymphatic vessels in the regressing CL. Unexpectedly, LYVE-1 antibody reacted with 3β-HSD positive cells. LYVE-1 antibody binds to hyaluronan receptor on the lymphatic endothelial cells [18]. We are unaware of any reports that luteal cells express hyaluronan receptor. Therefore, we checked the specificity of the LYVE-1 antibody for the lymphatic endothelial cells and the specificity of the 3β-HSD antibody for luteal cells using ovarian hilus sections and mid CL sections (Fig. S1). We confirmed that the antibodies were specific to each target cell. Thus, the bovine luteal cells seem to express hyaluronan receptor. However, the role of the hyaluronan receptor in luteal cells is unclear.

Many yellow cells were found in the lymphatic fluid drained from the ovaries with a regressing CL. Similar yellow cells were dissociated from the regressing CL by collagenase (Fig. S2). These findings suggest that the regressing CL was the source of the yellow cells. These yellow cells positively reacted with 3β-HSD antibody. Luteal cells contain lipid droplets for steroid hormone synthesis [19]. Staining with Bodipy (493/503), a lipid droplets-staining regent [17], confirmed that most of the 3β-HSD positive cells found in the lymphatic fluid stored lipid droplets in their cytoplasm. Their nuclei were mostly at the edge of the cytoplasm. The nuclei of mature white adipocytes containing many lipid droplets, are also located at the edge of the cytoplasm [20]. The luteal cells of the regressing CL, which secrete only a small amount of P4, store lipid droplets in their cytoplasm [21], [22]. These findings suggest that 3β-HSD positive cells with many lipid droplets originated from the regressing CL. The diameters of most 3β-HSD positive cell in lymphatic fluid were 15–20 µm. In the luteal cells in suspension, the large luteal cells had diameters of 23–57 µm and the small luteal cells had diameters of 12.5–23 µm [19]. Based on previous findings [17], [19], [20]–[22] and our present results, the 3β-HSD positive cells in the lymphatic vessels seem to be small luteal cells drained from the CL.

If the steroidogenic cells found in lymphatic fluid are small luteal cells, a question remains why large luteal cells do not flow out into the lymphatic vessels. A possible explanation is that only the large luteal cells might selectively disappear from the CL by apoptosis and phagocytosis or other mechanisms. Further studies are needed to identify the steroidogenic cells drained from the ovary.

In the regressing CL, the microvasculature disappears, but the arterioles with smooth muscle remain [23]. Similarly, the large lymphatic vessels with smooth muscle remain within the regressing CL after the lymphatic capillaries disappear [8]. Luteal cells in the CL might be carried to large lymphatic vessels via an unknown pathway. Further studies are needed to understand how luteal cells enter the lymphatic vessels.

The number of the immune cells in the bovine CL is reported to increase during luteolysis [7]. However, we are unaware of any reports on the number of immune cells in lymphatic fluid drained from the CL. The number of these cells might also increase during luteolysis. In fact, in the present study, the number of 3β-HSD negative cells (which may be immune cells) increased during luteolysis, and the number of 3β-HSD positive cells increased during luteolysis as well. However, since the ratio of 3β-HSD positive cells increased during luteolysis, the number of 3β-HSD positive cells seems to increase more than that of 3β-HSD negative cells.

In the present study, the percentage of 3β-HSD positive cells in the lymphatic fluid was higher at days 22–24 and days 26–28 after ovulation than at other luteal stages, indicating that the outflow of steroidogenic cells to lymphatic vessels increases after the start of the next estrous cycle. It has been reported that bovine CL tissue does not disappear completely before the next ovulation, and that the regressing CL from the previous estrous cycle remains about at 20% of its volume compared with the size of the mid CL [3]. Indeed, the regressing CL was observed on the surface of the ovaries during days 22–28 after ovulation in the present study. Therefore, the CL seems to disappear completely from the ovaries around days 26–28 after ovulation. In this study, the ratio of 3β-HSD positive cells was less at days 26–28 than at days 22–24. These findings suggest that the possible outflow of steroidogenic cells from the CL to the lymphatic vessels starts at the regressing luteal stage (days 19–21) and continues until around day 30 after ovulation. The outflow of steroidogenic cells via lymphatic vessels appears to engage in complete disappearance of the CL from the ovary. Interestingly, some 3β-HSD positive cells were found in lymphatic fluid drained from the ovary with the pregnant CL, and the ratio of these cells was less than 10%. The ratio of 3β-HSD positive cells was similar to that at days 8–12 (mid luteal stage) and 15–17 (late luteal stage). It is unclear why steroidogenic cells were drained from the active CL before the start of structural luteolysis. A possible explanation is that cells are drained from active tissue as cell turnover.

It takes 4–5 days to reduce the size of the CL after spontaneous PGF2α secretion from the uterus [3]. On the other hand, the volume of the CL decreased to less than half of its original size 24 h after PGF2α administration on Day 10 post ovulation [6]. However, the number of macrophages observed within the regressing CL during spontaneous and PGF2α-induced luteolysis is almost same [7]. Hence, the mechanism underlying the rapid structural luteolysis induced by PGF2α administration is unclear. We speculate that the number of luteal cells drained from the CL is greater during luteolysis induced by PGF2α administration than during spontaneous luteolysis. Further *in vivo* studies are needed to test this hypothesis.

**Figure 5:**
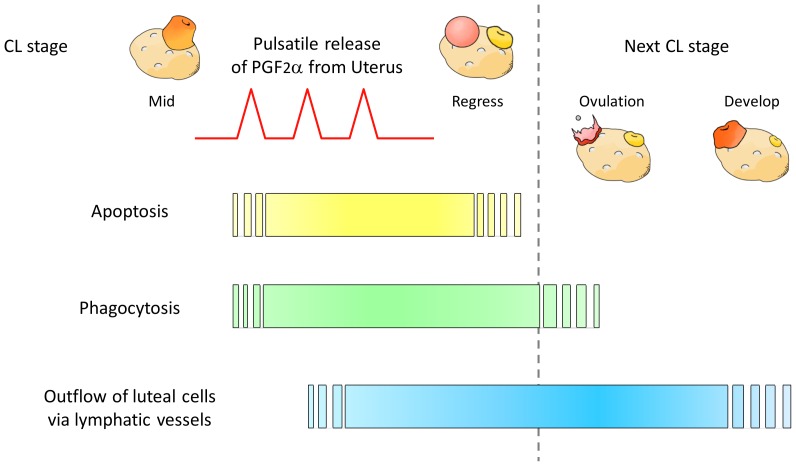
Possible mechanisms of structural luteolysis via lymphatic vessels. Bovine spontaneous luteolysis is caused by PGF2α from the uterus. During structural luteolysis, luteal cells are killed by apoptosis, and macrophages simultaneously infiltrate into the CL to remove luteal cells by phagocytosis. Immediately after starting phagocytosis, luteal cells start flowing into the lymphatic vessels. Luteolysis proceeds to a phase of next ovulation. After ovulation, the outflow of the luteal cells continues, and ensures the definitive disappearance of the CL tissue from the ovary.

Our results show that live steroidogenic cells are in the lymphatic vessels drained from the CL. These findings suggest that structural luteolysis involves not only apoptosis of luteal cells and phagocytosis, but also an outflow of luteal cells from the CL to the lymphatic vessels. This mechanism may ensure the definitive disappearance of the CL tissue during structural luteolysis. Possible mechanisms of structural luteolysis via the lymphatic vessels are shown in Figure 5.

## Supporting Information

Figure S1 **Specificities of LYVE-1 and 3β-HSD antibodies.** A section of ovarian hilus was incubated with LYVE-1 antibody, which stains lymphatic endothelial cells, and a section of the mid CL was incubated with 3β-HSD antibody, which stains luteal cells. A: Black arrowheads show LYVE-1 antibody positive cells, indicating lymphatic vessels. LYVE-1 antibody did not react with vascular endothelial cells (yellow arrowheads). Vascular vessels were identified by erythrocytes in the vessels (blue arrowhead). B: Negative control for confirming specificity of LYVE-1 antibody. C: Luteal cells in the mid CL were stained by 3β-HSD antibody (red arrowheads). D: Negative control for confirming specificity of 3β-HSD antibody. All bars, 50 µm. These results indicate that the antibodies used in this study worked well.
(TIF)

Figure S2 **Yellow cells in lymphatic fluid and in regressing CL.** To reveal the origin of the yellow cells in the lymphatic fluid drained from the ovary, we enzymatically isolated the cells of regressing CL. A: A regressing CL (at days 26–28 after ovulation; black arrowhead) was used. B: Yellow cells were dissociated from regressing CL by collagenase (red arrowheads). C: Similar yellow cells were found in lymphatic fluid drained from the ovary with this regressing CL (red arrowheads). All scale bars, 20 µm. The above results support our hypothesis that the source of the yellow cells in the lymphatic fluid was the regressing CL. In the present study, we showed that the yellow cells express 3β-HSD and contain lipid droplets.
(TIF)
