# Supplementary figures and images for: Lymphatic Involvement in the Disappearance of Steroidogenic Cells from the Corpus Luteum during Luteolysis
Source: PLoS One. 2014 Feb 20;9(2):e88953. doi: 10.1371/journal.pone.0088953 (PMC3930584; doi:10.1371/journal.pone.0088953)

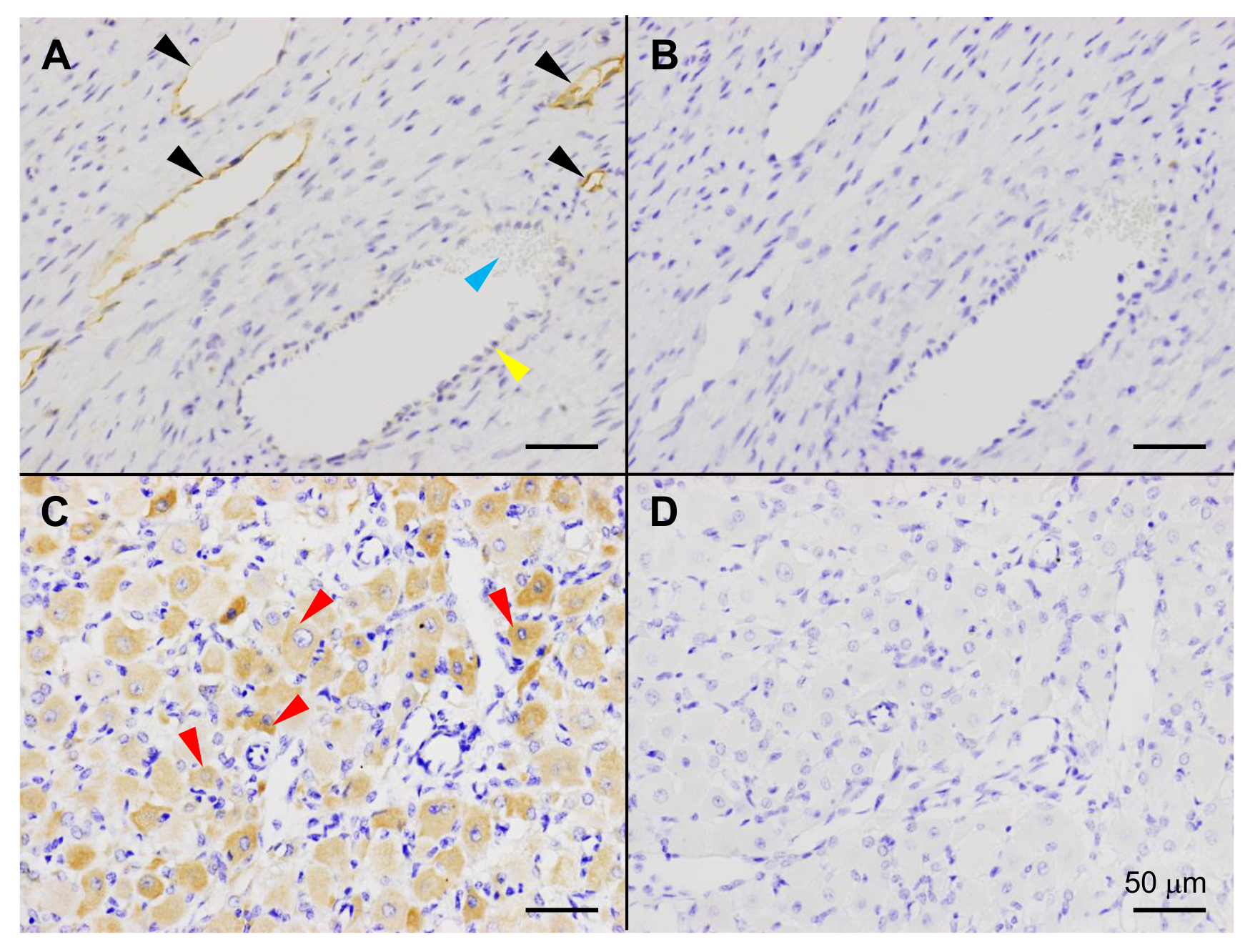

Supplement: Figure S1 — Specificities of LYVE-1 and 3β-HSD antibodies. A section of ovarian hilus was incubated with LYVE-1 antibody, which stains lymphatic endothelial cells, and a section of the mid CL was incubated with 3β-HSD antibody, which stains luteal cells. A: Black arrowheads show LYVE-1 antibody positive cells, indicating lymphatic vessels. LYVE-1 antibody did not react with vascular endothelial cells (yellow arrowheads). Vascular vessels were identified by erythrocytes in the vessels (blue arrowhead). B: Negative control for confirming specificity of LYVE-1 antibody. C: Luteal cells in the mid CL were stained by 3β-HSD antibody (red arrowheads). D: Negative control for confirming specificity of 3β-HSD antibody. All bars, 50 µm. These results indicate that the antibodies used in this study worked well. (TIF) [file pone.0088953.s001.tif]

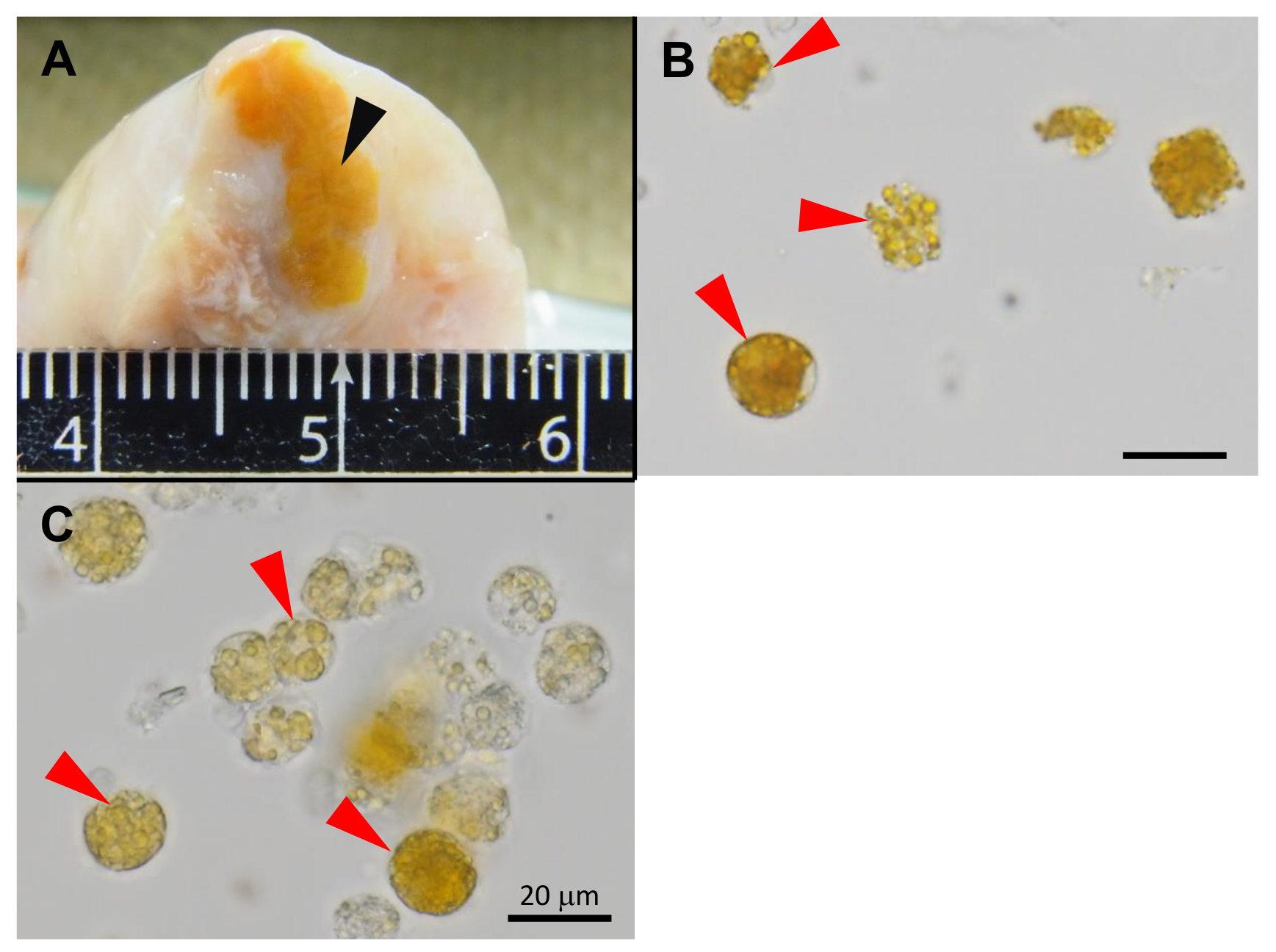

Supplement: Figure S2 — Yellow cells in lymphatic fluid and in regressing CL. To reveal the origin of the yellow cells in the lymphatic fluid drained from the ovary, we enzymatically isolated the cells of regressing CL. A: A regressing CL (at days 26–28 after ovulation; black arrowhead) was used. B: Yellow cells were dissociated from regressing CL by collagenase (red arrowheads). C: Similar yellow cells were found in lymphatic fluid drained from the ovary with this regressing CL (red arrowheads). All scale bars, 20 µm. The above results support our hypothesis that the source of the yellow cells in the lymphatic fluid was the regressing CL. In the present study, we showed that the yellow cells express 3β-HSD and contain lipid droplets. (TIF) [file pone.0088953.s002.tif]
